# A Comparison of Surgical Auditory Nerve Response and Speech Outcomes in Patients with Post-meningitic Deafness and Without Cochlear Osteogenesis Who Underwent Cochlear Implantation

**DOI:** 10.7759/cureus.5650

**Published:** 2019-09-13

**Authors:** Mohammed Alshaikh, Asmaa Alahmadi, Mohammed Albedry, Abdulmajeed Alharbi, Saad Alenzi, Rawan Almahyawi, NoorJehan Mansouri, Mohammad Albaqeyah, Abdullah Alamri, Amani A Alharbi, Ahmad Aldajani

**Affiliations:** 1 ENT and Cochlear Implant Center, Royal Commission Hospital, Jubail, SAU; 2 Otolaryngology Head and Neck Surgery Department, King Fahad Hospital, Jeddah, SAU; 3 Faculty of Medicine, King Abdulaziz University Hospital, Jeddah, SAU; 4 Otolaryngology Department, University of Jeddah, Jeddah, SAU

**Keywords:** cochlear implantation, meningitis, sensory neural deafness, cochlear sclerosis, speech recognition threshold

## Abstract

Objective

Patients with post-meningitis deafness remain challenging candidates for cochlear implantation (CI) which can be difficult due to fibrosis or ossification of the inner ear, and their outcomes remain doubtful. We assessed the surgical and audiological outcomes of CI in patients with profound sensorineural hearing loss caused by meningitis and compared those outcomes to patients without cochlear ossification.

Methods

This retrospective cross-sectional study was carried out at King Fahad General Hospital, Jeddah, Saudi Arabia. Among 246 patients who underwent cochlear implantation, 13 patients with post-meningitic deafness were identified (Group 1). A matched control group, including patients with deafness due to other causes who did not have cochlea osteogenesis, was selected (Group 2). For all patients, data were collected from medical records, including surgical and audiological outcomes.

Results

Sclerosis of the cochlea was high in Group 1 (46.2%). There were no postoperative surgical complications in either group. Responses of the auditory nerve action potential obtained through auditory response telemetry (ART) or the neural response telemetry (NRT) were recorded. There was no significant difference between the two groups regarding the intraoperative and the postoperative ART or NRT at selected electrodes representing the entire cochlea. Likewise, no significant difference regarding the speech recognition test (SRT) was detected.

Conclusions

Cochlear implantation is a safe procedure without surgical complications in post-meningitis patients. Furthermore, early CI in children was associated with favorable outcomes in terms of preservation of the auditory nerve response, restoration of speech discrimination, and recognition to levels comparable to patients with deafness due to other causes. Early audiological assessment in meningitis patients is recommended to identify hearing loss and eventually to offer CI.

## Introduction

Cochlear implantation (CI) is the best-accepted modality to rehabilitate adults and children with severe to profound sensory neural hearing loss [1]. A cochlear implant is an effective procedure that can provide aid to the development of auditory perception, favoring the acquisition of the linguistic processes, especially in children, which will undoubtedly contribute to all aspects of development. Surgical and functional outcomes of the auditory performance vary among implantees. Variable factors influence the outcome, e.g., the age of the patient, and the etiology of sensory neural hearing loss (SNHL) (whether congenital or acquired) affecting the prognosis [2]. Moreover, the duration of deafness and psychosocial conditions could eventually affect CI outcomes [3].

One of the most prevalent acquired etiologies of SNHL is bacterial meningitis (BM), with estimates ranging from 60% to 90% of all cases of acquired SNHL in children [4]. Approximately 10% of survivors of BM in developed countries are left with permanent SNHL [5], which is caused mainly by direct bacterial damage to the organ of Corti due to inflammation, subsequent fibrosis, and potential ossification [6]. Cochlear ossification following BM has been identified in a large percentage of patients with profound deafness resulting from BM [7]. The frequency and severity of the ossification vary according to the offending organism, with pneumococcal meningitis at the top of the list of the highest incidence of ossification, followed by Neisseria meningitidis [8]. Cochlear ossification is seen as early as two months after the onset of meningitis and is described in up to 70% of the ears [9]. Rapid obliteration of the cochleas due to osteoneogenesis is the main cause of difficulty in cochlear implantation.

Application of cochlear implants in survivors of BM is a challenging procedure due to fibrosis or ossification of the inner ear. Insertion of an electrode may be only partial or even impossible [10]. Therefore, patients with post-meningitis deafness remain challenging candidates for CI and their outcomes remain doubtful. However, all studies recommend early referral of those children for audiological assessment and fast cochlear implantation before ossification becomes established. The objectives of this study were to identify the surgical and audiological outcomes of CI in patients with profound sensorineural hearing loss caused by BM and to compare those outcomes to the outcomes in patients without cochlear ossification.

## Materials and methods

Study design

This retrospective, cross-sectional study was carried out at King Fahad General Hospital, Jeddah, Saudi Arabia. Among the 246 patients who underwent cochlear implantation, 13 patients with post-meningitic deafness were identified (Group 1) after reviewing the medical records. A control group (Group 2) was selected to be matched in number (13 patients), age, age of implantation, and duration of deafness. This group included patients with deafness due to causes other than meningitis, and they did not have cochlea osteogenesis.

Data collection

For all patients, data were collected from medical records, including demographic and medical information. In addition, responses of the auditory nerve action potential obtained via auditory response telemetry (ART) or neural response telemetry (NRT) and the speech recognition threshold (ART) defined as the minimum intensity in decibels at which a patient can understand 50% of spoken words were evaluated. Moreover, the surgical outcomes during and after the operation were characterized.

Data entry and statistical analysis

A Microsoft® Excel® spreadsheet (Microsoft® Corp., Redmond, WA) was utilized for data entry. Data were statistically analyzed using the IBM Statistical Package for the Social Sciences (SPSS) version 20.0 (IBM SPSS Statistics, Armonk, NY). The Chi-square test was used to estimate the relationship between categorical variables. When more than 20% of cells had an expected count of less than 5, Fisher’s exact test was used. For continuous data, the Shapiro Wilk test was done for testing their distribution, and the independent t-test was used for comparison. Significance was adopted at p < 0.05 for interpretation of the test results.

## Results

Most of the patients with post-meningitic deafness in Group 1 were males (92.3%) compared to seven males (53.8%) in Group 2. The median age of patients in Group 1 was non-significantly higher than in Group 2 (six years versus five years, respectively). The median age of implantation was exactly similar in both groups (four years). The gender distribution, age of patients, and age of implantation in both groups are shown in Table 1.

**Table 1:** Demographic Data of the Studied Cases IQR: interquartile range

			Groups			P-value
			Group 1 (N = 13)	Group 2 (N = 13)	Total (N = 26)	
Gender	Female	N	1	6	7	.073
		%	7.7%	46.2%	26.9%	
	Male	N	12	7	19	
		%	92.3%	53.8%	73.1%	
Age (years)	Minimum		1.00	2.00	1.00	1.00
	Maximum		30.00	36.00	36.00	
	Median		6.00	5.00	5.50	
	IQR		3.00 - 7.00	3.00 - 7.00	3.00 - 7.00	
	Mean rank		13.46	13.54		
Age at the time of surgery (years)	Minimum		1.00	1.00	1.00	.88
	Maximum		30.00	36.00	36.00	
	Median		4.00	4.00	4.00	
	IQR		3.00 - 6.00	3.00 - 6.00	3.00 - 6.00	
	Mean rank		13.23	13.77		

In the majority of cases, the operation was done on the right side (100% and 92.3% for Groups 1 and 2, respectively). For both groups, the prosthesis was of the MED-EL (MED-EL Medical Electronics, Innsbruck, Austria) type in the majority of the studied cases (61.5%), and Cochlear™ Nucleus® (Cochlear Ltd., Sydney, Australia) for the remainder.

In both groups, the vast majority of cases did not have a past history of medical illness. There was no significant difference between both groups regarding a family history of SNHL or the presence of parental consanguinity. The medical information of the studied groups are shown in Table 2.

**Table 2:** Medical Information of the Studied Cases *significant SNHL: sensory neural hearing loss

		Groups						P-value
		Group 1 (N = 13)		Group 2 (N = 13)		Total (N = 26)		
		N	%	N	%	N	%	
Medical illness	Yes	1	7.7%	1	7.7%	2	7.7%	1.00
	No	12	92.3%	12	92.3%	24	92.3%	
Chronic disease	None	11	84.6%	12	92.3%	23	88.5%	1.00
	Bronchial Asthma	1	7.7%	0	0.0%	1	3.8%	
	Epilepsy	1	7.7%	0	0.0%	1	3.8%	
	Hypothyroidism	0	0.0%	1	7.7%	1	3.8%	
Family history	Yes	5	38.5%	5	38.5%	10	38.5%	1.00
	No	8	61.5%	8	61.5%	16	61.5%	
First-degree relatives	Yes	4	30.8%	5	38.5%	9	34.6%	1.00
	No	9	69.2%	8	61.5%	17	65.4%	
Preoperative degree of hearing loss	Profound SNHL	13	100.0%	7	53.8%	20	76.9%	.015*
	Severe SNHL	0	0.0%	4	30.8%	4	15.4%	
	Unknown	0	0.0%	2	15.4%	2	7.7%	
Preoperative otitis media effusion (OME)	Yes	0	0.0%	2	15.4%	2	7.7%	.480
	No	13	100.0%	11	84.6%	24	92.3%	
OME- medical treatment	Yes	0	0.0%	1	7.7%	1	3.8%	1.00
	No	13	100.0%	12	92.3%	25	96.2%	
OME- surgical treatment	Yes	0	0.0%	1	7.7%	1	3.8%	1.00
	No	13	100.0%	12	92.3%	25	96.2%	
Program number	3.00	0	0.0%	2	15.4%	2	7.7%	.744
	4.00	4	30.8%	5	38.5%	9	34.6%	
	5.00	5	38.5%	4	30.8%	9	34.6%	
	6.00	2	15.4%	2	15.4%	4	15.4%	
	7.00	1	7.7%	0	0.0%	1	3.8%	
	8.00	1	7.7%	0	0.0%	1	3.8%	
Speech at last visit	.00	1	7.7%	0	0.0%	1	3.8%	.929
	70.00	1	7.7%	1	7.7%	2	7.7%	
	90.00	3	23.1%	2	15.4%	5	19.2%	
	95.00	3	23.1%	3	23.1%	6	23.1%	
	100.00	5	38.5%	7	53.8%	12	46.2%	
Computed tomography (CT) scan	Non-sclerotic	8	61.5%	12	100.0%	20	80.0%	.039*
	Sclerotic	5	38.5%	0	0.0%	5	20.0%	
Side of operation	Right	13	100.0%	12	92.3%	25	96.2%	1.00
	Left	0	0.0%	1	7.7%	1	3.8%	
Prosthesis	Cochlear™ Nucleus®	5	38.5%	5	38.5%	10	38.5%	1.00
	MED-EL	8	61.5%	8	61.5%	16	61.5%	

In terms of the preoperative degree of hearing loss, all cases in Group 1 had a profound degree of SNHL (100%) as compared to Group 2 (53.8%) and with a significant difference between both groups (p = .015).

All cases in Group 1 had no preoperative otitis media effusion (OME), which was present in only two cases in Group 2; one of them was treated medically, but the other one was managed surgically.

Regarding computed tomography (CT) scan findings, no cases in Group 2 showed cochlear sclerosis compared to five cases (38.5%) in Group 1 with a significant difference between both groups (p = .039).

In both groups, the number of programming sessions to reach a performance plateau was four to five times in the majority of cases with no significant difference (p = 0.12). There was no significant difference between both groups in regards to the maximum achieved speech discrimination.

There was no significant difference found between both groups regarding each of the previously mentioned items. The prenatal, perinatal, delivery, and neonatal information of the studied groups is shown in Table 3.

**Table 3:** Prenatal, perinatal, delivery, and neonatal information of the studied cases. CS: caesarean section; NA: not applicable; NICU: newborn intensive care unit

		Groups						Fisher’s exact test
		Group 1 (N = 13)		Group 2 (N = 13)		Total (N = 26)		
		N	%	N	%	N	%	P-value
Perinatal jaundice	No	13	100.0%	13	100.0%	26	100.0%	NA
NICU	Yes	3	23.1%	0	0.0%	3	11.5%	.220
	No	10	76.9%	13	100.0%	23	88.5%	
Antibiotic usage	Yes	4	30.8%	0	0.0%	4	15.4%	.096
	No	9	69.2%	13	100.0%	22	84.6%	
Preterm baby	Yes	1	7.7%	0	0.0%	1	3.8%	1.00
	No	12	92.3%	13	100.0%	25	96.2%	
Low birth weight	Yes	1	7.7%	0	0.0%	1	3.8%	1.00
	No	12	92.3%	13	100.0%	25	96.2%	
Syndromic baby	No	13	100.0%	13	100.0%	26	100.0%	NA
Pre-eclampsia	No	13	100.0%	13	100.0%	26	100.0%	NA
Maternal fever or infection	Yes	0	0.0%	1	7.7%	1	3.8%	1.00
	No	13	100.0%	12	92.3%	25	96.2%	
Type of delivery	Vaginal	12	92.3%	11	84.6%	23	88.5%	1.00
	CS	1	7.7%	2	15.4%	3	11.5%	

Sclerosis of the cochlea was present in 46.2% in Group 2 compared to none in Group 1. In both groups, there were no postoperative complications in any of the patients. The operative and postoperative findings in the studied groups are shown in Table 4.

**Table 4:** Surgical Outcomes of the Studied Groups CSF: cerebrospinal fluid; NA: not applicable *Significant

		Groups						Fisher’s exact test
		Group 1 (N = 13)		Group 2 (N = 13)		Total (N = 26)		
		N	%	N	%	N	%	P-value
Postoperative facial weakness	No	13	100.0%	13	100.0%	26	100.0%	NA
Postoperative meningitis	No	13	100.0%	13	100.0%	26	100.0%	NA
Postoperative device failure	No	13	100.0%	13	100.0%	26	100.0%	NA
Wound infection	No	13	100.0%	13	100.0%	26	100.0%	NA
Wound dehiscence	No	13	100.0%	13	100.0%	26	100.0%	NA
Hematoma or seroma	No	13	100.0%	13	100.0%	26	100.0%	NA
Facial twitches	No	13	100.0%	13	100.0%	26	100.0%	NA
Postoperative vertigo	No	13	100.0%	13	100.0%	26	100.0%	NA
Postoperative tinnitus	No	13	100.0%	13	100.0%	26	100.0%	NA
Postoperative acute otitis media	No	13	100.0%	13	100.0%	26	100.0%	NA
CSF gusher	No	13	100.0%	13	100.0%	26	100.0%	NA
Intraoperative bleeding	No	13	100.0%	13	100.0%	26	100.0%	NA
Operative findings	Sclerotic, full insertion of electrode	6	46.2%	0	0.0%	6	23.1%	.015*
	Smooth, full insertion of electrode	7	53.8%	13	100.0%	20	76.9%	

Five electrodes were selected: the most apical one, the most basal one, and three in-between electrodes. These electrodes were selected to represent the entire cochlea. There were no significant differences between the two groups with regards to the intraoperative and the postoperative ART or NRT. The intraoperative and postoperative ART/NRT of the studied groups are shown in Table 5.

**Table 5:** Intraoperative and Postoperative Auditory Nerve Response (ART/NRT) in the Studied Groups ART: auditory nerve response; NRT: neural response telemetry; SD: standard deviation

		Groups			T-test
		Group 1 (N = 13)	Group 2 (N = 13)	Total (N = 26)	P-value
Intraoperative ART/NRT					
The most apical electrode	Minimum	165.00	157.00	157.00	.395
	Maximum	200.00	199.00	200.00	
	Mean ± SD	182.46 ± 13.09	178.00 ± 13.20	180.23 ± 1 3.08	
Three different electrodes between the most basal and most apical electrodes	Minimum	158.00	157.00	157.00	.479
	Maximum	200.00	199.00	200.00	
	Mean± SD	181.85±15.79	177.69±13.60	179.77±14.59	
	Minimum	167.00	175.00	167.00	.144
	Maximum	200.00	197.00	200.00	
	Mean± SD	190.23±10.69	184.69±7.75	187.46±9.58	
	Minimum	163.00	169.00	163.00	.176
	Maximum	225.00	201.00	225.00	
	Mean± SD	195.23±18.68	186.62±12.16	190.92±16.05	
The most basal electrode	Minimum	160.00	168.00	160.00	.160
	Maximum	217.00	202.00	217.00	
	Mean± SD	196.00±18.96	186.69±13.32	191.35±16.74	
Post-operative ART/ NRT					
The most apical electrode	Minimum	160.00	154.00	154.00	.398
	Maximum	181.00	187.00	187.00	
	Mean± SD	165.77±6.47	168.77±10.72	167.27±8.81	
Three different electrodes between the most basal and most apical electrodes	Minimum	157.00	152.00	152.00	.565
	Maximum	188.00	196.00	196.00	
	Mean ± SD	167.92±9.91	170.46±12.14	169.19±10.94	
	Minimum	157.00	160.00	157.00	.943
	Maximum	189.00	190.00	190.00	
	Mean ± SD	175.00±12.42	175.31±9.10	175.15±10.67	
	Minimum	158.00	160.00	158.00	.614
	Maximum	199.00	193.00	199.00	
	Mean ± SD	179.00±15.20	176.38±10.47	177.69±12.86	
The most basal electrode	Minimum	155.00	158.00	155.00	.382
	Maximum	199.00	193.00	199.00	
	Mean ± SD	175.54±14.95	171.08±10.16	173.31±12.73	

In both groups, the intensity ranged from 40 - 50 with a median of 45. The non-significant difference between the studied groups regarding the SRT test (p > 0.05) are shown in Table 6.

**Table 6:** Comparison of Speech Recognition Threshold (SRT) in the Studied Groups IQR: interquartile range

					Mann-Whitney test
		Group 1 (N = 13)	Group 2 (N = 13)	Total	P-value
SRT	Minimum - Maximum	40.0 - 50.0	40.0 - 50.0	40.0 - 50.0	.336
	Median	45.0	45.0	45.0	
	IQR	40.0 - 45.0	45.0 - 50.0	40.0 - 50.0	
	Mean rank	12.04	14.96		

## Discussion

Cochlear implantation after post-meningitic deafness showed favorable outcomes. This is what our present study demonstrated - promising and similar outcomes after CI in terms of the auditory nerve response, speech discrimination, and surgical complications among patients with deafness due to meningitis compared to those with deafness due to other reasons. Furthermore, when audiological performances were evaluated, speech recognition and the comprehensive ability of the patients to the spoken words were comparable in both groups. Although CI required some special consideration in patients with deafness due to meningitis, it was found to be a safe procedure without surgical complications.

Similar results were obtained by Francis et al. [11] and Nikolopoulos et al. [12] in children who underwent CI due to deafness resulting from meningitis compared to children with different causes of deafness. Another study in accordance with our conclusions on children but with longer follow-up for three years after CI surgery concluded that auditory capacity and speech performance were comparable in children with post-meningitic deafness and those undergoing implantation for other reasons [13]. Likewise, speech recognition was assessed by Wellman and colleagues in children who underwent CI in the prelingual period [14]. No significant differences were mentioned between those children with post-bacterial meningitis deafness and those who had profound SNHL due to various other causes. A long-term favorable audiological outcome was reported by Tokat and colleagues [15]. 

Our results are in contrast with El-Kashlan et al. who found that although prelingually deafened children with post-meningitic hearing loss and ossified cochleae received significant benefit from cochlear implants, their performance was frequently lacking in comparison with children with non-ossified cochlea [16]. Consistent with El-Kashlan et al., a recent study done by Ikeya et al. [10] on adults demonstrated that patients with post-meningitic deafness benefitted significantly from cochlear implantation; however, the audiological outcomes were still hard to predict in some cases, especially in the presence of ossification [10]. In the one year follow-up after utilizing the device, assessment of speech recognition revealed poorer results in cochlear implant recipients with hearing loss due to bacterial meningitis than those with hearing loss due to other causes who used the device for the same period of time [17]. In an Iranian study, a survey was done for the assessment of CI outcomes in the form of auditory and speech abilities in post-meningitis deaf children and revealed that outcomes were not the same as in non-meningitis deaf children. However, most of the studies confirmed that CI was the only and, in most cases, the best way to help these children [18].

One of the factors influencing the success rate of CI is the time gap between deafness and surgery; early implantation is an essential mandatory factor for the development of good results. In our center, patients with post-meningitic SNHL were identified and underwent CI early. This early identification and intervention might have led to the observed better outcomes. 

Cochlear implantation is the standard treatment of profound post-meningeal SNHL for its benefit of regaining auditory capability, as well as speech performance. It is known that the number of electrodes activated postoperatively is a crucial factor for the presence of good audiological results. The existence of cochlear ossification hinders the full insertion of electrodes in conventional cochlear implants, leading to a worse audiological result when compared with non-ossified cochleas [6]. Although cochlear ossification was evidenced at surgery in 46.2% of patients in the current study, full insertion of electrodes was done in all cases. This is another factor that could explain the non-significant differences between the studied groups. However, it should be noted that other factors, such as the employed rehabilitation method and the stimulation received, influence the results in children with prelingual hearing loss [19].

As long as the neurons in the spiral ganglion and the more central neuronal networks remain intact and well-functioning, you can expect excellent results might be achieved with CI in patients suffering from post-meningeal SNHL [20]. Therefore, meticulous follow-up of patients with meningitis and early detection of cochlear ossification is an important influential factor for surgical success. In our study, the preoperative CT scan was used to detect sclerosis. However, it was observed that CT imaging has limitations for identification of the early stages of cochlear ossification in the basal turn, being surpassed by magnetic resonance imaging (MRI), which is able to identify the stage of fibrosis of the perilymphatic space prior to ossification [6]. 

This study has the advantage of quantifying the performance of the patients and the ability to understand spoken speech. This poses more valid and applied outcomes. However, the presence of a small sample size could be considered as a limitation.

## Conclusions

Cochlear implantation was found to be a safe procedure without surgical complications in patients with deafness due to meningitis. Early CI in children with deafness due to meningitis was associated with favorable outcomes in terms of preservation of auditory nerve response and restoration of speech discrimination and recognition to levels comparable to patients with deafness due to congenital or other causes. It is, therefore, highly recommended to do an early referral and audiological assessment in meningitis patients as soon as possible to identify hearing loss and eventually to offer CI.
